# Human Miscarriage Is Associated With Dysregulations in Peripheral Blood-Derived Myeloid Dendritic Cell Subsets

**DOI:** 10.3389/fimmu.2019.02440

**Published:** 2019-10-15

**Authors:** Stefanie Ehrentraut, Karoline Sauss, Romy Neumeister, Lydia Luley, Anika Oettel, Franziska Fettke, Serban-Dan Costa, Stefanie Langwisch, Ana Claudia Zenclussen, Anne Schumacher

**Affiliations:** ^1^Health Campus Immunology, Infectiology and Inflammation (GC-I^3^), Experimental Obstetrics and Gynecology, Medical Faculty, Otto-von-Guericke University, Magdeburg, Germany; ^2^Gynecologic Ambulance, Haldensleben, Germany; ^3^University Women's Clinic, Otto-von-Guericke University, Magdeburg, Germany

**Keywords:** peripheral blood dendritic cells, plasmacytoid dendritic cells, myeloid dendritic cells, regulatory T cells, human chorionic gonadotropin, placenta factors, fetal tolerance, pregnancy

## Abstract

Dendritic cells (DC) are critically involved in decisions related to the acceptance or rejection of the foreign fetal antigens by the maternal immune system. However, particularly for human peripheral blood DCs (PBDC), available literature is rather inconsistent and the factors regulating these cells are ill-defined. Here, we investigated the phenotype and functionality of different human PBDC subsets during normal and pathologic pregnancies and studied an involvement of human chorionic gonadotropin (hCG) in PBDC regulation. Peripheral blood samples were obtained from normal pregnant women in all three trimesters, from first trimester miscarriage patients and from healthy non-pregnant women. Samples were analyzed for plasma hCG levels, for regulatory T (Treg) cell numbers, for frequencies of total and mature plasmacytoid (PDC) and myeloid (MDC1 and MDC2) PBDC subsets and for their cytokine secretion. *In vitro* assays, culturing PDC, MDC1 or MDC2 in the presence of two trophoblast cell lines, placenta explant supernatants or two hCG preparations were performed. The Treg-inducing capability of hCG- or non-hCG-treated stimulated MDC1 was assessed. Total and mature MDC1 and MDC2 frequencies increased during the first and second trimester of normal pregnancy, respectively. Miscarriage was associated with a reduced MDC1 and an increased MDC2 activation profile. PDC were not altered neither during normal pregnancy progression nor during miscarriage. *In vitro*, the culture of isolated PBDC subsets in the presence of placenta-derived factors impaired the maturation of MDC1 and differentially affected PDC maturation. An inhibitory effect on MDC1 and PDC maturation was also proven for the urine-derived hCG preparation. Finally, we observed a Treg cell elevation during early normal pregnancy that was not present in miscarriages. Stimulated MDC1 induced Treg cells *in vitro*, however, hCG was not involved in this process. Our findings suggest that during normal pregnancy PBDC subsets are differentially regulated dependent on gestational age. Miscarriage seems to be associated with dysregulations in the myeloid PBDC subsets and with disturbances in Treg cell frequencies. Moreover, our results propose an interdependency between MDC1 and Treg cells during early pregnancy. hCG, although shown to impair MDC1 maturation, does not seem to be a key regulator of PBDC alterations during pregnancy.

## Introduction

Pregnancy is characterized by finely regulated immunological adaptions that allow the persistence of the foreign fetal antigens in the maternal womb. Notably, since Schmorl and colleagues in 1893 identified for the first time fetal cells at various maternal tissue sites (1), it became obvious that not only local immune cell populations residing directly at the fetal-maternal interface but also immune cell types circulating in the periphery get in direct contact to fetal structures and are able to react toward them. Thus, pregnancy-driven immunological adjustments take place locally and peripherally and dysregulations in uterine as well as circulating immune responses may interfere with normal pregnancy progression. Several studies implicated immune modulatory properties of placenta-derived factors suggesting that the fetal tissue itself regulates its surrounding environment (2). These factors include among others cytokines, growth factors and hormones and were reported to educate local and peripheral immune cells in such a way that they contribute to fetal tolerance and growth (3–9). Thereby, peripheral immune cell populations reportedly adapt their phenotype and functionality so to enter the fetal-maternal interface as “fetal-friendly” cells. However, the precise mechanisms how this is realized during pregnancy are still under investigation.

As one of the first immune cell types encountering fetal antigens, dendritic cells (DCs) possess the capacity to induce either immunity or tolerance toward the fetus (10). Their behavior highly depends on the maturation state and cytokine secretion pattern (10). In the human decidua, the presence of predominantly myeloid DCs possessing an immature phenotype and secreting anti-inflammatory cytokines was associated with fetal tolerance (11–14), while a mature DC phenotype could be associated with various pregnancy complications (15–18). By contrast, data referring to the distribution and the phenotype of peripheral blood DC (PBDC) subsets during normal and pathologic pregnancies are rather inconsistent which might be due to the different DC characterization strategies applied in the past (19–22). Moreover, most of the studies divided the total PBDC pool into two major subsets, one lymphoid, and one myeloid subset. However, in 2000 Dzionek and colleagues described the existence of three clearly defined PBDC subsets. Based on the expression of specific blood DC antigens (BDCA), the authors classified the total PBDC pool into one plasmacytoid (PDC) and two myeloid (MDC1 and MDC2) subsets (23). Later on, the Nomenclature Committee of the International Union of Immunological Societies approved this classification (24). Interestingly, the presence of the BDCA-defined DC subsets seems to be not restricted to the peripheral blood as Ban and colleagues were able to detect all three subsets in the human decidua during early normal pregnancy. Decidual BDCA-1^+^ MDC1 and BDCA-3^+^ MDC2 expressed low levels of the maturation markers CD86 and CD80 confirming an immature phenotype of local DCs during normal pregnancy (25). This finding gives rise to speculations about the origin of these cells. On the one hand, it can be assumed that BDCA-1^+^ MDC1 and BDCA-3^+^ MDC2 are part of the DC pool resident in the uterus and regulated locally once pregnancy arised. On the other hand, it can be hypothesized that both DC subsets immigrate from the peripheral blood stream into the fetal-maternal interface. In both cases, placenta-derived factors may contribute to the local regulation as well as to DC immigration (26–28). Zhao and colleagues suggested a participation of human placenta-produced factors in the differentiation process from blood-derived monocytes into decidual DCs. The authors further confirmed a tolerogenic phenotype of these differentiated DCs (29). Additionally, placental factors reaching the circulation may provoke alterations of PBDCs directly in the periphery. The pregnancy hormone human chorionic gonadotropin (hCG) may represent a good candidate to fulfill this function as it represents one, if not the first signal, provided by the fetal tissue to the mother and can be detected already 6–8 days following fertilization in the blood stream (30). Furthermore, there is evidence that hCG has the capability to modulate human PBDCs (20, 31, 32).

Here, we aimed to investigate the phenotype and functionality of PDC, MDC1, and MDC2 derived from human peripheral blood during normal and pathologic pregnancies. Moreover, we addressed a potential regulation of all PBDC subsets by placenta-derived factors with a specific focus on hCG.

## Methods

### Sampling and Ethical Approval

Peripheral blood samples were obtained from healthy non-pregnant woman within the luteal phase of their menstrual cycle as well as from normal pregnant woman in all three trimesters and from first trimester miscarriage patients. Placental tissue from normal pregnant women and miscarriage patients was obtained by curettage during surgery. Sampling of blood and placental tissue was realized by physicians of the University Women's Clinic in Magdeburg, Germany after all subjects were informed in detail and gave their written consent. This study was carried out in accordance with the recommendations of ethic guidelines defined by the ethics board at the University of Magdeburg with written informed consent from all subjects. All subjects gave written informed consent in accordance with the Declaration of Helsinki. The protocol was approved by the ethics board at the University of Magdeburg (study 28/08). Characteristics of all study subjects are displayed in Table 1.

**Table 1:** Patient characteristics.

	**Total number**	**Week of pregnancy**	**Age**
***Ex vivo*** **analyses**			
Non-pregnant women	*n =* 12	N/A	24.82 ± 4.41
Normal pregnant women (I. Trimester)	*n =* 20	10.30 ± 1.39	25.95 ± 5.06
Normal pregnant women (II. Trimester)	*n =* 14	19.21 ± 3.97	28.36 ± 5.45
Normal pregnant women (III. Trimester)	*n =* 18	34.28 ± 3.38	30.06 ± 3.22
Miscarriage patients	*n =* 20	8.65 ± 1.11	30.70 ± 6.01
***In vitro*** **Assays**			
Non-pregnant women	*n =* 6	N/A	28.50 ± 4.15
Normal pregnant women (I. Trimester)	*n =* 16	9.81 ± 1.70	30.00 ± 5.97
Miscarriage patients (I. Trimester)	*n =* 16	8.69 ± 0.98	31.50 ± 4.85

### Determination of hCG Isoforms in Plasma and Placenta Supernatants by ELISA Analysis

After tissue collection, 500 mg of placental tissue (explants) was cultured in 1 ml of RPMI 1640 (Thermo Fisher Scientific, Germany) supplemented with 3% of charcolized fetal bovine serum (FBS, PAN-Biotech, Germany) and 1% penicillin/streptomycin (Thermo Fisher Scientific, Germany) for 24 h. Afterwards, placenta explant supernatants were collected and analyzed for the concentration of either regular hCG, free β-hCG or hyperglycosylated hCG (H-hCG) by enzyme-linked immunosorbent assay (ELISA). The concentrations of all hCG isoforms were evaluated in the plasma fraction of all blood samples. Regular and free β-hCG were determined using kits from DRG systems, Germany whereas H-hCG concentrations were evaluated using a kit from My Biosource, USA. All steps were performed according to the manufacturer's instructions.

### Isolation of MDC1, MDC2, or PDC From PBMCs

The cellular fraction from all blood samples was used to isolate peripheral blood mononuclear cells (PBMCs) by density gradient centrifugation using Ficoll-Paque^TM^ (GE Healthcare, Sweden) under sterile conditions. Afterwards, MDC1, MDC2, or PDC were separately isolated from PBMCs of non-pregnant and normal pregnant women (I. trimester) as well as from miscarriage patients (I. trimester) by magnetic activated cell sorting. The following isolations kits from Miltenyi Biotec, Germany were applied: MDC1 (CD1c Dendritic Cell Isolation Kit, human); MDC2 (CD141 MicroBead Kit, human); and PDC (CD304 MicroBead Kit, human). All steps were performed under sterile conditions following the instructions given. Purities of isolated MDC1, MDC2 and PDC were above 95, 45, and 85%, respectively. After isolation, all PBDC subsets were cultured for 24 h in RPMI 1640 supplemented with 50 μM β-mercaptoethanol (Sigma Aldrich, Germany), 10% FBS (Biochrom, Germany) and 1% penicillin/streptomycin (dendritic cell medium; DCM).

### Cytometric Bead Array (CBA) Analysis of Cytokine Secretion by MDC1, MDC2, and PDC

5 × 10^4^ isolated MDC1, MDC2, or PDC from either normal pregnant women or miscarriage patients in their first trimester of pregnancy were cultured in DCM for 48 h. Following, cell supernatants were collected and analyzed for the levels of IL-1β, IL-6, IL-8, IL-10, and TNF by CBA using the TH1/TH2 Cytokine Kit from BD Biosciences, Germany. All steps were performed according to the instructions provided by the manufacturer. Measurements were conducted by using a 4-color FACSCalibur™ flow cytometer (BD Biosciences, Germany) and analyses were performed using FCAPArray software (BD Biosciences, Germany).

### Assessment of PBDC Maturation Under Different Culture Conditions Involving Trophoblast Cell Lines, Placental Explant Supernatants or Purified hCG Preparations

For the following experiments, MDC1, MDC2, or PDC were isolated from PBMCs derived from healthy non-pregnant women in the luteal phase of their menstrual cycle. The maturation of all three PBDC subsets was induced by the addition of 10 ng/ml lipopolysaccharide (LPS) to the DCM. In each experimental setting, the maturation state was assessed after 24 h by determining the numbers of CD83-, CD86-, or HLA-DR-expressing MDC1, MDC2, or PDC. The appropriate gating strategy is displayed in Supplementary Figure 1.

In the first set of experiments, 1 × 10^4^ MDC1, MDC2, or PDC were co-cultured with 3 × 10^4^ JEG-3 cells (hCG-secreting human choriocarcinoma cell line) or SWAN-71 cells (non hCG-secreting human immortalized extravillious cytotrophoblast cell line). Therefore, both trophoblast cell lines were plated 1 day before starting the co-cultures to allow adherence to the culture plate. JEG-3 and SWAN-71 cells were cultured in Dulbecco's modified eagle medium (DMEM, Invitrogen, Germany) supplemented with 10% FBS, 1% penicillin/streptomycin, 100 nM MEM non-essential amino acids (Invitrogen, Germany), 1 mM sodium pyruvate (Sigma-Aldrich, Germany), and 10 mM hepes (Biochrom, Germany). Both cell lines were cultured at 37°C and 5% CO_2_.

In the second set of experiments, 1 × 10^4^ MDC1, MDC2, or PDC were cultured in placental explant supernatants enriched with DCM in a ratio 1:1. Placental explants were derived from normal pregnant women or miscarriage patients in their first trimester of pregnancy.

In the third set of experiments, 1 × 10^4^ MDC1, MDC2, or PDC were treated with either 100IU/ml recombinant hCG (rhCG, Ovitrelle, Merck, Germany) or 250 IU/ml urine-derived hCG (uhCG, Sigma, Germany). The concentration of uhCG was chosen according to physiological hCG levels found in normal pregnant women during the first trimester (25-288IU/ml during gestation weeks 9–12 according to the *American Pregnancy Association*) and rhCG concentration was chosen according to concentrations used for rhCG and other recombinant gonadotropins in previous studies (33, 34).

### Assessment of Treg Cell Generation After Co-culture With hCG- or Non hCG-Treated MDC1

MDC1 were isolated from non-pregnant women, stimulated with 10 ng/ml LPS and cultured in the absence or presence of hCG (100 mIU/ml rhCG or 250IU/ml uhCG) for 24 h as described above. Afterwards, 1 × 10^4^ MDC1 were co-cultured with 1 × 10^4^ CD4^+^CD25^−^ T cells in DCM containing 10 ng/ml recombinant IL-2, 1 μg/ml anti-CD3 and 5 μg/ml anti-CD28 for another 24 h. CD4^+^CD25^−^ T cells were obtained from the same donors who provided the MDC1 by using the human CD4^+^CD25^+^ Regulatory T Cell Isolation Kit (Miltenyi Biotec, Germany). Frequencies of CD4^+^CD25^high^Foxp3^+^ Treg cells were determined by flow cytometry using a 4-color FACSCalibur™ flow cytometer. The appropriate gating strategy is displayed in Supplementary Figure 2.

### Flow Cytometry Analysis

To estimate the frequencies of total and mature MDC1, MDC2, and PDC within PBMCs from non-pregnant and normal pregnant women as well as from miscarriage patients, PBMCs were stained for distinct surface markers defining the different DC subsets and for the maturation markers CD83, CD86, and HLA-DR. Additionally, Treg cell frequencies were determined in all samples. Therefore, PBMCs were stained for the extracellular markers CD4 and CD25 as well as for the intracellular marker Foxp3. Briefly, PBMCs were washed in PBS containing 1% bovine serum albumin (BSA) and 0.1% sodium azide (flow cytometry (FC) buffer). Afterwards, staining for extracellular markers (1:100 antibody dilutions) was performed for 30 min at 4°C in the dark. Following another washing step in FC buffer, cells were fixed overnight using the fixation/permeabilization set from Thermo Fisher Scientific, Germany. For intracellular staining, PBMCs were washed in permeabilization buffer and then incubated for 30 min at 4°C in the dark in the staining solution (1:200 antibody dilution). After washing in permeabilization buffer, cells were resuspended in FC buffer and measured using a 4-color FACSCalibur flow cytometer. The appropriate gating strategy is displayed in Supplementary Figure 3. MDC1, MDC2, PDC, and T cells obtained from *in vitro* culture experiments were stained accordingly. The following antibodies were used: APC-conjugated anti-human CD303 (PDC, clone: AC144), APC-conjugated anti-human CD1c (MDC1, clone: AD5-8E7) or APC-conjugated anti-human CD141 (MDC2, clone: AD5-14H12); all purchased from Miltenyi Biotec, Germany. PE-Cy7-conjugated anti-human CD83 (clone: HB15e), FITC-conjugated anti-human CD86 (clone: FUN-1), PE-conjugated anti-human HLA-DR (clone: G46-6), FITC-conjugated anti-human CD4 (clone: OKT4), PerCp-Cy5.5-conjugated anti-human CD25 (clone: BC96), and AF647-conjugated anti-human Foxp3 (clone: 259D/C7); purchased from BioLegend, eBiosciences and BD Biosciences, Germany, respectively.

### Data Analysis and Statistics

Data analysis and presentation were performed with GraphPad Prism 7.0 software (Statcon, Germany). Data are displayed as means plus standard deviation (S.D.) or standard error of the mean (S.E.M.). The total number of replicates is indicated in the figure legends for each specific data set. All data sets were tested for normality by using the Shapiro-Wilk test and parametric or non-parametric tests were used accordingly. Normal distributed data sets were analyzed by using One- or Two-Way-ANOVA followed by Tukey's or Dunn‘s multiple comparison test. Not normal distributed data sets were analyzed by applying the Mann-Whitney-*U*-test. For *in vitro* culture experiments, controls (LPS alone) were set as “1” and experimental groups (trophoblast co-cultures, placenta explant supernatants and hCG preparations) were calculated as fold change to controls.

## Results

### Pregnancy Provokes Alterations in the Number of Total and Mature Myeloid PBDC Subsets

**Figure 1:**
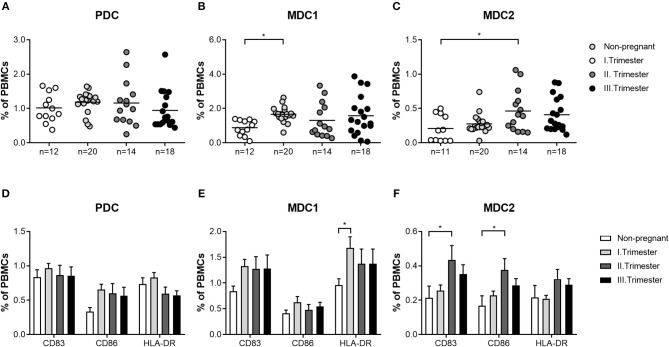
The frequencies of total and mature MDC1 and MDC2 are altered depending on gestational age. PBMCs were isolated from normal pregnant women in all trimesters (*n* = 14–20) as well as from healthy non-pregnant women (*n* = 12) and stained for markers defining the distinct PBDC subsets and for the maturation markers CD83, CD86, and HLA-DR. The total frequencies of **(A)** PDC, **(B)** MDC1, and **(C)** MDC2 as well as the frequencies of mature **(D)** PDC, **(E)** MDC1, and **(F)** MDC2 were determined by flow cytometry. Statistical analysis was performed using One- or Two-Way-ANOVA followed by Tukey's multiple comparison test. *p ≤ 0.05.

First, we wondered whether PBDC subsets would change depending on gestational age during normal pregnancy progression. All subsets represented minor populations within total PBMCs (PDC: 0.25–2.64%; MDC1: 0.07–3.87%; MDC2: 0.03–1.06%). Frequencies of total and mature PDC from normal pregnant women of all trimesters were not altered when compared to non-pregnant women (Figures 1A,D). However, we observed an increase in the frequencies of total MDC1 and MDC2 during the first and second trimester in normal pregnant women, respectively, as compared to non-pregnant individuals (Figures 1B,C). Accordingly, HLA-DR-expressing MDC1 as well as CD83 and CD86-expressing MDC2 were elevated at the same gestational ages (Figures 1E,F).

### Miscarriage Is Associated With an Altered Profile of Both Myeloid PBDC Subsets

**Figure 2:**
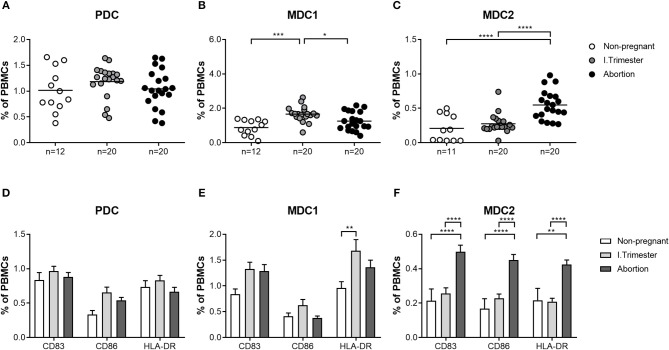
The frequencies of total and mature MDC1 and MDC2 are altered in miscarriages. PBMCs were isolated from normal pregnant women and miscarriage patients in the first trimester (*n* = 20) as well as from healthy non-pregnant women (*n* = 12) and stained for markers defining the distinct PBDC subsets and for the maturation markers CD83, CD86, and HLA-DR. The total frequencies of **(A)** PDC, **(B)** MDC1, and **(C)** MDC2 as well as the frequencies of mature **(D)** PDC, **(E)** MDC1, and **(F)** MDC2 were determined by flow cytometry. Statistical analysis was performed using One- or Two-Way-ANOVA followed by Tukey's multiple comparison test. *p ≤ 0.05, **p ≤ 0.01, ***p ≤ 0.001, ****p ≤ 0.0001.

Next, we sought to determine whether similar alterations in both myeloid PBDC subsets could be observed in miscarriage patients. We found no augmentation of total MDC1 frequencies in patients suffering from abortions when compared to non-pregnant women and a significant diminution as compared to normal pregnant women (Figure 2B). Moreover, frequencies of HLA-DR-expressing MDC1 were reduced in miscarriage patients when compared to normal pregnant women (Figure 2E). However, this did not reach statistical significance. Notably, frequencies of total and mature MDC2 were significantly elevated in miscarriage patients as compared to non-pregnant and normal pregnant women (Figures 2C,F). This included CD83-, CD86- and HLA-DR-expressing MDC2 (Figure 2F). As for PDC, no significant changes could be detected in miscarriage patients, neither to normal pregnant women nor to non-pregnant individuals (Figures 2A,D).

### Miscarriage Is Associated With a Disturbed Cytokine Secretion Capacity of the MDC1 Subset

**Figure 3:**
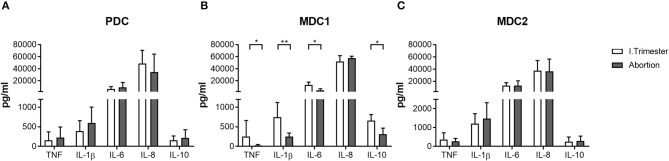
MDC1 from miscarriages showed an altered cytokine profile. PDC, MDC1, or MDC2 were isolated from PBMCs of normal pregnant women and miscarriage patients in the first trimester (*n* = 4–5). All PBDC subsets were cultured for 48 h. Afterwards, cell culture supernatants were analyzed for the levels of different cytokines by CBArray. The concentration of TNF, IL-1β, IL-6, IL-8, and IL-10 produced by **(A)** PDC, **(B)** MDC1, and **(C)** MDC2 is displayed. Statistical analysis was performed using the Mann-Whitney-*U*-test. *p ≤ 0.05, **p ≤ 0.01.

Based on our findings, showing altered frequencies of total and mature MDC1 and MDC2 in miscarriage patients, we were curious whether both myeloid subsets would also possess a disturbed cytokine secretion profile. Therefore, we separately assessed the capacity to secrete cytokines of all PBDC subsets after their isolation from normal pregnant women or miscarriage patients. In agreement with our previous results, PDC from miscarriage patients secreted equal amounts of pro- and anti-inflammatory cytokines as the ones from normal pregnant women (Figure 3A). MDC1 from miscarriage patients had a significant reduced capacity to secrete IL-1β, IL-6, IL-10, and TNF (Figure 3B), while MDC2 from miscarriage patients did not behave differentially from MDC2 of normal pregnant women (Figure 3C). However, we have to admit that the purity of the MDC2 isolation was rather poor.

### Miscarriage Is Associated With Reduced Peripheral and Local Levels of Different hCG Isoforms

As our results revealed changes during early normal pregnancy and predominantly in the MDC1 subset that could not be observed in pathologic pregnancies, we wondered whether placenta-derived factors might be responsible for the observed alterations. Here, we particularly focused on the pregnancy hormone hCG, known to be highly elevated during the first trimester. Analyses of plasma samples as well as placental explant supernatants exhibited significant diminished levels of regular hCG in plasma samples from miscarriage patients when compared to normal pregnant women but not in placental explant supernatants (Table 2). Even more interestingly, we found significant reduced levels of two other hCG isoforms, namely free β-hCG and H-hCG, in the plasma and the placenta supernatants from miscarriage patients (Table 2). Correlation analyses between all hCG isoforms and all PBDC subsets demonstrated a negative correlation between regular hCG or H-hCG and MDC2 but no correlation between free β-hCG and MDC2 (data not shown). Moreover, no correlations could be proven for all hCG isoforms and MDC1 or PDC (data not shown).

**Table 2:** Miscarriage is associated with reduced levels of hCG isoforms.

	**regular hCG (mIU/ml)**	**free β-hCG (pg/ml)**	**H-hCG (nmol/L)**
**Plasma**			
I. Trimester	112855 ± 106610	43.63 ± 37.91	38.21 ± 43.43
Abortion	43840 ± 46906^*^	23.57 ± 22.56^*^	12.70 ± 5.43^**^
**Placenta explants**			
I. Trimester	175.60 ± 216.10	97.99 ± 42.48	198.40 ± 261.40
Abortion	252.30 ± 261.20	53.67 ± 54.53^*^	64.69 ± 92.57^*^

*Plasma and placenta explants from normal pregnant women and miscarriage patients in the first trimester (n = 15–20) were analyzed for the levels of different hCG isoforms by ELISA. Statistical analysis was performed using the Mann-Whitney-U-test*.

* *p < 0.05 vs. I. Trimester*;

** *p < 0.01 vs. I. Trimester*.

### Placenta-Derived Factors Differentially Regulate the Maturation of PBDC Subsets

**Figure 4:**
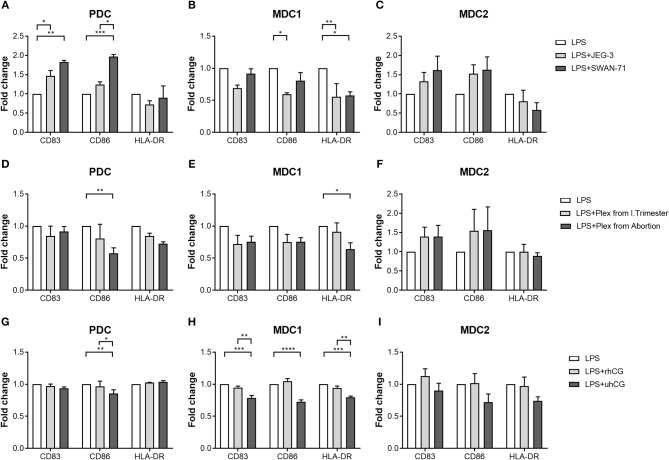
PDC, MDC1 and MDC2 are differentially affected by placenta-derived factors. PDC, MDC1, or MDC2 were isolated from PBMCs of healthy non-pregnant women and cultured in the presence of **(A–C)** JEG-3 or SWAN-71 trophoblast cell lines (*n* = 3) **(D–F)**, supernatants of placental explants (Plex; *n* = 4) or **(G–I)** two hCG preparations [100 mIU/ml recombinant hCG (rhCG); 250IU/ml urine-derived (uhCG)]. The frequencies of mature **(A,D,G)** PDC, **(B,E,H)** MDC1, and **(C,F,I)** MDC2 were determined by flow cytometry. Statistical analysis was performed using Two-Way-ANOVA followed by Tukey's multiple comparison test. *p ≤ 0.05, **p ≤ 0.01, ***p ≤ 0.001, ****p ≤ 0.0001.

To determine whether placenta-derived factors and particularly hCG might be involved in PBDC alterations, we first co-cultured PDC, MDC1 or MDC2 with two different trophoblast cell lines and evaluated the effect on the maturation process. We chose one hCG-secreting trophoblast cell line (JEG-3) and one non hCG-secreting trophoblast cell line (SWAN-71). The presence of hCG-secreting JEG-3 cells contributed to an increase of CD83-expressing PDC but impaired an elevation of CD86- and HLA-DR-expressing MDC1 (Figures 4A,B). Non hCG-secreting SWAN-71 cells favored an augmentation of CD83- and CD86-expressing PDC and hampered an increase of HLA-DR-expressing MDC1 (Figures 4A,B). None of the cell lines significantly affected the maturation process of MDC2 (Figure 4C).

Secondly, we decided to culture each PBDC subsets with supernatants from placental explants derived from either normal pregnant women or miscarriage patients. Here, in addition to our co-cultures with the trophoblast cell lines, we studied a potential influence of soluble factors derived from primary trophoblast cells mimicking a more physiologic scenario. Besides, it is important to mention that placental explant supernatants contain a mixture of all hCG isoforms whereas JEG-3 cells almost exclusively secrete H-hCG. Unfortunately, most of our primary trophoblast cultures lose the ability to secrete hCG in culture and were therefore not suitable for these kind of experiments. Supernatants from miscarriage patients significantly hampered an augmentation of CD86-expressing PDC and HLA-DR-expressing MDC1 (Figures 4D,E), whereas supernatants from normal pregnant women had no effect on PDC and MDC1 (Figures 4D,E). Moreover, maturation of MDC2 was not influenced by the addition of placental explant supernatants (Figure 4F).

Thirdly, as our data were rather inconsistent with regard to a potential effect of hCG, we cultured PDC, MDC1, or MDC2 in the presence of two hCG preparations. We chose an rhCG preparation containing only intact regular hCG molecules and an uhCG preparation containing the broad variety of all hCG isoforms. We observed an impaired elevation of CD86-expressing PDC as well as CD83-, CD86-, and HLA-DR-expressing MDC1 when uhCG was added to the cultures (Figures 4G,H), whereas the presence of rhCG did not affect the maturation of PDC and MDC1 (Figures 4G,H). In line with our previous results, MDC2 maturation was not affected at all (Figure 4I). However, again we would like to point out that our MDC2 isolations suffered from a poor purity.

### hCG Does Not Enhance MDC1-Induced Treg Generation

**Figure 5:**
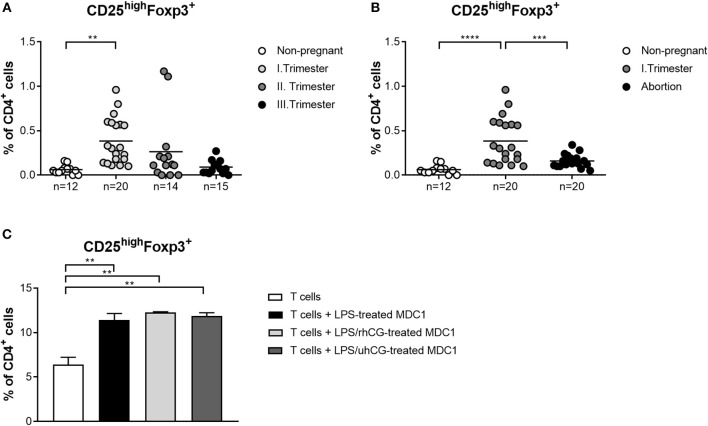
MDC1 cells are able to induce Treg cells that are reduced in miscarriages. PBMCs were isolated from normal pregnant women in all trimesters (*n* = 14–20), from miscarriage patients in the first trimester (*n* = 20) as well as from healthy non-pregnant women (*n* = 12) and stained for Treg cell markers. Treg frequencies are shown for **(A)** normal pregnancy progression and for **(B)** miscarriage patients compared to normal and non-pregnant women. Moreover, MDC1 were isolated from non-pregnant women (*n* = 3), stimulated with 10 ng/ml LPS in the presence or absence of 100 mIU/ml recombinant hCG (rhCG) or 250IU/ml urine-derived hCG (uhCG) for 24 h and then co-cultured with CD4^+^CD25^−^ T cells derived from the same donors for another 24 h. **(C)** Treg frequencies were determined by flow cytometry. Statistical analysis was performed using One-Way-ANOVA followed by Dunn's multiple comparison test. **p ≤ 0.01, ***p ≤ 0.001, ****p ≤ 0.0001.

Own previous studies identified hCG as a factor driving Treg induction during early pregnancy (35). However, it is still a matter of debate through which pathways hCG mediates its Treg-inducing properties. Here, we wondered whether an interdependency between Treg and PBDCs during early pregnancy exists and if hCG might represent the intermediate factor between both immune cell types. Therefore, we determined the frequencies of Treg during normal pregnancy progression as well as during miscarriage. CD4^+^CD25^high^Foxp3^+^ Treg frequencies were significantly elevated in first trimester normal pregnant women when compared to non-pregnant women and declined during the second and third trimester (Figure 5A). Miscarriage patients had significantly reduced Treg frequencies as compared to normal pregnant women (Figure 5B). Notably, normal pregnancy-associated Treg alterations are in line with changes observed for MDC1 (Figures 1B, 5A). Moreover, miscarriage was characterized by lower Treg and MDC1 frequencies suggesting an interrelation between these two cell populations. To follow up this idea, we tested whether MDC1 possessed the ability to induce Treg *in vitro*. We pre-treated MDC1 with rhCG or uhCG to study a potential involvement of hCG in MDC1-mediated Treg induction. We confirmed the ability of MDC1 to induce Treg generation. However, pre-treatment with either rhCG or uhCG did not further enhance Treg numbers (Figure 5C).

## Discussion

Pregnancy can be considered as a remarkable challenge for the maternal immune system where a variety of immunological adaptions has to go hand in hand to guarantee the survival of the semi-allogeneic fetus. Hereby, not only local immune cell populations residing at the fetal-maternal interface but also immune cell types circulating through lymphoid tissues and the peripheral blood are aware of the foreign fetal antigens. Thus, pregnancy-driven immunological regulations take place in the proximity as well as in the distance of the fetal tissues and it is suggested that the fetus itself contributes to these regulatory processes by expressing and secreting immune modulating factors.

DCs are one of the major immune cell populations that are affected by pregnancy-driven alterations and on their part function as key regulators for other immune cells types. There is accumulating evidence that during normal human pregnancy progression myeloid DCs are the predominant DC population, locally as well as peripherally (11, 19, 20). Moreover, human decidual DCs adopt a tolerogenic profile by expressing an exclusive set of markers and possessing a reduced T cell stimulatory capacity (11, 12). PBDCs also undergo changes in their phenotype and functionality throughout normal human pregnancy. However, several studies presented different outcomes (19–22, 36) that might be partly explained by the combination of markers used to characterize the various PBDC subsets. Furthermore, the majority of the previous studies did not separately examine the frequencies of MDC1 and MDC2 but rather focused on one myeloid PBDC population. This kind of analysis may mask potential gestational-dependent alterations within distinct myeloid PBDC subsets.

Our current findings propose significant augmentations of total MDC1 and MDC2 during the first and second trimester of normal pregnancy, respectively, while total PDC remained unchanged at all trimesters. Likewise, we found elevations of mature myeloid PBDCs during the first and second trimester while the number of mature PDC was not altered. These observed gestational age-related changes in the number and functionality of PBDCs seem to be relevant to finely regulate the degree of alloreactive immune reactions. On the one hand, PBDCs have to be alert to systemic infections at any time while on the other hand ensure tolerance toward the fetal alloantigens. To overcome this paradoxical situation, it was proposed that PBDCs pass through an incomplete activation process during normal pregnancy enabling them to present fetal alloantigens without provoking overwhelming anti-fetal immune reactions. Moreover, there is evidence that PBDCs are involved in the induction of acquired thymic tolerance and it was suggested that incompletely activated PBDCs promote tolerance to fetal alloantigens by educating thymic T cells (19). Notably, the extent of DC activation seems to be decisive for pregnancy outcome as an over- or under-activation of PBDCs was associated with pregnancy complications such as pre-eclampsia, intrauterine growth retardation or miscarriage (18, 22). Our own findings support this idea, as we were able to demonstrate a significant lower number of mature MDC1 in miscarriage patients compared to normal pregnant women. Moreover, these cells had a reduced capacity to secrete cytokines suggesting an overall diminished potential to stimulate T cells. Our data suggest that also an underactivation of specific PBDC subsets may provoke fetal demise. Limited activation of MDC1 may result in a reduced potential to induce Treg cells that at first glance seems to be contradictory, as it is believed that predominantly DCs being in an immature or semi-mature state force Treg generation (37). However, Banerjee and colleagues proved that human myeloid DCs matured with a cocktail of inflammatory cytokines had the highest capability to induce Treg cells. Even myeloid DCs matured in the presence of LPS induced Treg cells to a greater extent than immature DCs (38). Our own data revealed that LPS-stimulated MDC1 supported the generation of Treg cells *in vitro*. Hence, we propose that during early stages of normal pregnancy, activated MDC1 enhance Treg cells in the periphery whereas underactivated MDC1 may lack the capacity for an adequate Treg induction. Our findings provide another explanation for the observed early Treg increment that is fundamental for fetal tolerance induction as reported earlier (39, 40) and again confirmed in our current study. Interestingly, MDC2 showed a completely different pattern when compared to MDC1 underlying the need to separately study the different myeloid PBDC subsets during pregnancy. Mature MDC2 were significantly augmented in miscarriage patients as compared to normal pregnant women. We assume that MDC1 and MDC2 overtake different functions at different gestational ages and that an over or underrepresentation of each subset at other pregnancy time points may be deleterious for pregnancy. According to a study by Hayashi and colleagues, MDC1 can be seen as TH1-promoting cells while MDC2 are rather TH2-promoting cells (41). As normal pregnancy begins with TH1-related immune responses (peri-implantation phase), changes to TH2-related immune responses (mid-gestation) and finally shifts back to TH1-related immune responses at the end of pregnancy (labor induction) (42), our data suggest a critical role for MDC1 during the first trimester and for MDC2 during the second trimester. Here, at specific pregnancy periods both myeloid PBDC subsets may keep the delicate balance between pro- and anti-inflammatory immune reactions that are required for maintenance of a normal pregnancy.

After confirming pregnancy-associated alterations in the PBDCs and particularly in the MDC1 subset, we wondered whether placental-derived factors might provoke these changes. Previous studies proposed various effects of placental factors on DCs from different origins (26, 43–46). Our own data revealed no remarkable effect of soluble factors derived from normal primary trophoblast cells on all PBDC subsets. In contrast, supernatants from miscarriage trophoblast cells and co-culture of PBDCs with the trophoblast cell lines JEG-3 and SWAN-71 profoundly affected the number of mature PDC and MDC1. Among the molecules suggested to participate in placenta-driven PBDC modulation are: HLA-G, TGF-β, and indoleamine-2,3 dioxygenase (46) as well as pregnancy hormones. The latter ones, due to their autocrine and paracrine modes of action, can affect resident cells at the fetal-maternal interface and distant cells in the periphery (47, 48). Segerer and colleagues confirmed an inhibitory effect of hCG on DC maturation while estrogen and progesterone did not have any impact (32). Interestingly, Della Bella and colleagues, although not concentrating on hCG-mediated effects, suggested this hormone to be involved in the incomplete activation of PBDC during human pregnancy (19). In our current study, the presence of uhCG impaired the maturation of MDC1 and affected the frequency of mature PDC while rhCG did not. This only partly reflects our previous results where we found an inhibitory effect of hCG on the MDC1 subset that was mainly mediated by rhCG. However, in this study, we did not separately examine each PBDC subset and interdependencies between PDC, MDC1 and MDC2 could therefore not be excluded (31). A limiting factor in the present study was the purity of the isolated MDC2 that could be achieved by magnetic cell isolation. According to the manufacturer's instructions in the manual of this specific MDC2 isolation kit, some MDC1, PDC and monocytes are co-enriched with MDC2. Thus, we cannot completely exclude an influence of these “contaminating” immune cell populations on a potential hCG-mediated effect on MDC2 and conclusions derived from our MDC2 data have to be considered with caution.

In contrast to our data, Yoshimura and colleagues exhibited a stimulatory effect of hCG on myeloid and plasmacytoid PBDCs including the maturation, the cytokine secretion and the T cell stimulatory capacity (20). This shows that data referring to an hCG-mediated function on PBDCs during pregnancy are still inconsistent and further studies are needed to finally clarify this issue. Particularly, the PBDC characterization strategy has to be standardized to make studies comparable. Moreover, equivalent hCG preparations should be applied or at least a clear definition of the preparation used in each study should be provided. Our own investigations let assume that the immunological effects of rhCG or uhCG differ in some aspects and we suggest that this might be due to the different composition of both preparations. While rhCG preparations only contain intact molecules of regular hCG, uhCG preparations comprise intact and nicked variants of all hCG isoforms that can be physiologically found during pregnancy. These hCG isoforms have been shown to possess distinct functions. Regular hCG primarily promotes *corpus luteal* progesterone production, induces cytotrophoblast differentiation and contributes to uterine angiogenesis whereas free-β hCG exerts growth-promoting activity and blocks apoptosis (49). H-hCG, together with regular hCG promotes angiogenesis of the uterine vasculature and represents a key factor for trophoblast invasion (50). Notably, H-hCG was reported to activate the TGF-β receptor (51) implicating a potential pathway through which this isoform may regulate immune cell activity. Although our analysis of the different plasma hCG isoforms revealed that miscarriage patients suffer from reduced levels of regular hCG; free-β hCG and H-hCG as well as from altered MDC1 and MDC2 frequencies, based on our *in vitro* assays we do not believe that hCG is causative for the PBDC changes associated with miscarriage. However, we suggest that hCG has the potential to affect the phenotype of the distinct PBDC subsets.

In conclusion, our findings suggest that during normal pregnancy progression PBDC subsets are differentially regulated depending on gestational age. Miscarriage seems to be associated with dysregulations in the frequencies and functionality of the myeloid PBDC subsets as well as with disturbances in Treg frequencies. In the light of our results, we further propose the existence of an interdependency between MDC1 and Treg cells during early pregnancy. hCG, although shown to correlate negatively with MDC2 frequencies and to impair MDC1 maturation, does not seem to be a key regulator of PBDC alterations during pregnancy.

## Data Availability Statement

All datasets generated for this study are included in the manuscript/Supplementary Files.

## Ethics Statement

This study was carried out in accordance with the recommendations of ethic guidelines defined by the ethics board at the University of Magdeburg with written informed consent from all subjects. All subjects gave written informed consent in accordance with the Declaration of Helsinki. The protocol was approved by the ethics board at the University of Magdeburg (study 28/08).

## Author Contributions

SE and KS performed and analyzed experiments. AS designed and supervised experiments. SE and AS prepared figures, interpreted data, and wrote the manuscript. S-DC, RN, LL, AO, and FF provided human sample material. SL and AZ critically revised the manuscript and provided financial support.

### Conflict of Interest

The authors declare that the research was conducted in the absence of any commercial or financial relationships that could be construed as a potential conflict of interest.
