# Supplementary figures and images for: Human Miscarriage Is Associated With Dysregulations in Peripheral Blood-Derived Myeloid Dendritic Cell Subsets
Source: Front Immunol. 2019 Oct 15;10:2440. doi: 10.3389/fimmu.2019.02440 (PMC6803452; doi:10.3389/fimmu.2019.02440)

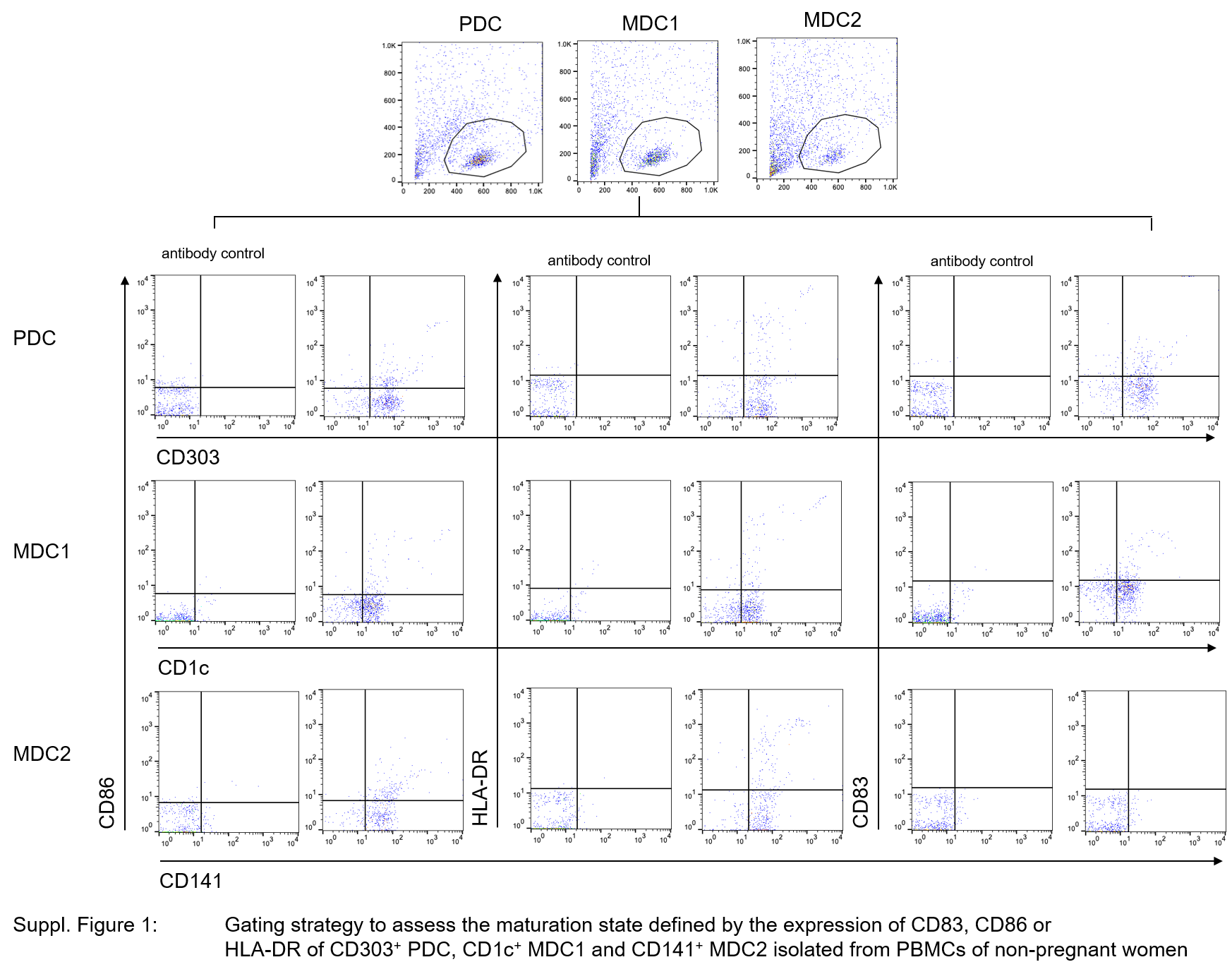

Supplement: Supplementary file 1 [file Image_1.TIF]

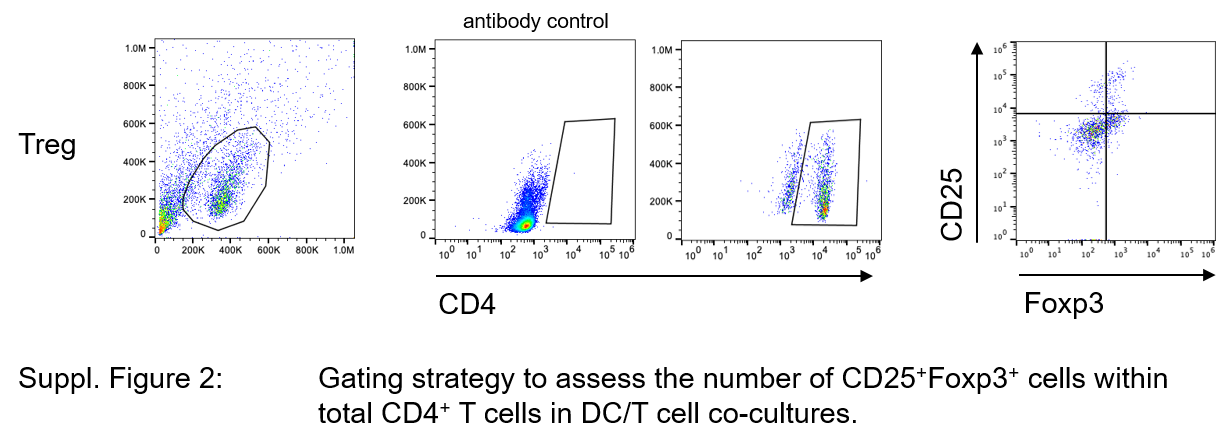

Supplement: Supplementary file 2 [file Image_2.TIF]

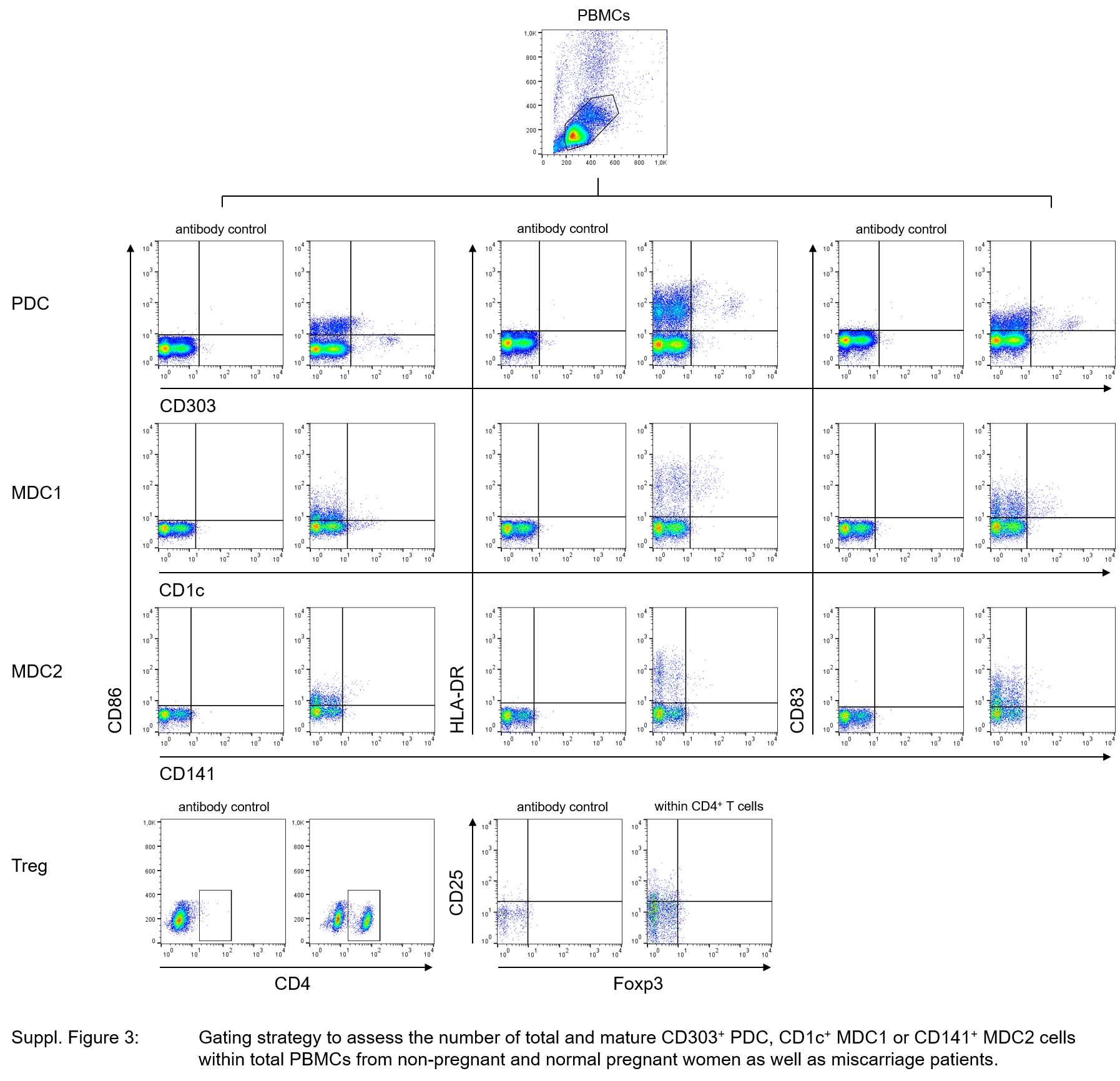

Supplement: Supplementary file 3 [file Image_3.TIF]
